# Association of the trajectory of plasma aldosterone concentration with the risk of cardiovascular disease in patients with hypertension: a cohort study

**DOI:** 10.1038/s41598-024-54971-4

**Published:** 2024-02-28

**Authors:** Xintian Cai, Shuaiwei Song, Junli Hu, Qing Zhu, Di Shen, Wenbo Yang, Huimin Ma, Qin Luo, Jing Hong, Delian Zhang, Nanfang Li

**Affiliations:** Hypertension Center of People’s Hospital of Xinjiang Uygur Autonomous Region, Xinjiang Hypertension Institute, NHC Key Laboratory of Hypertension Clinical Research, Key Laboratory of Xinjiang Uygur Autonomous Region “Hypertension Research Laboratory”, Xinjiang Clinical Medical Research Center for Hypertension (Cardio-Cerebrovascular) Diseases, No. 91 Tianchi Road, Ürümqi, 830001 Xinjiang China

**Keywords:** Adrenal gland diseases, Acute coronary syndromes, Hypertension, Vascular diseases, Calcification, Cardiovascular diseases, Metabolic syndrome, Risk factors

## Abstract

The purpose of this study was to determine the long-term pattern of plasma aldosterone concentration (PAC) trajectories and to explore the relationship between PAC trajectory patterns and cardiovascular disease (CVD) risk in patients with hypertension. Participants were surveyed three times between 2010 and 2016, and latent mixed modeling was employed to determine the trajectory of PAC over the exposure period (2010–2016). A Cox regression analysis was used to examine the association between PAC trajectory patterns and the risk of CVD (stroke and myocardial infarction). Hazard ratios (HRs) with corresponding 95% confidence intervals (CIs) were calculated and reported. During a median follow-up of 4.10 (3.37–4.50) years, 82 incident CVD cases (33 myocardial infarction cases and 49 stroke cases) were identified. Among all three PAC models, the high-stability PAC pattern exhibited the highest risk of CVD. After full adjustment for all covariables, HRs were 2.19 (95% CI 1.59–3.01) for the moderate-stable pattern and 2.56 (95% CI 1.68–3.91) for the high-stable pattern in comparison to the low-stable pattern. Subgroup and sensitivity analyses verified this association. The presence of a high-stable PAC trajectory pattern is associated with an elevated risk of CVD in hypertensive patients. Nevertheless, more studies are warranted to confirm these findings.

## Introduction

Cardiovascular disease (CVD) is the leading cause of mortality worldwide, posing a significant threat to human health^1^. In China, the burden of CVD is substantial, with 120.33 million individuals affected by CVD and 4.58 million CVD-related deaths recorded in 2019, placing strain on the healthcare system^2^. Hypertension, a preventable risk factor, plays a crucial role in the development of CVD and premature mortality globally^3^. Unfortunately, hypertension is highly prevalent in China and is projected to surge in the coming decades^4^. Among the Chinese population, hypertension represents the most significant risk factor for CVD^5^, accounting for 43% of CVD cases^2,6^. Therefore, the identification of high-risk individuals among hypertensive patients is crucial for alleviating the burden of CVD.

Plasma aldosterone concentration (PAC) is a notable and controllable risk factor for CVD that has a high degree of prognostic significance for the disease burden in the future^7–10^. Several studies have demonstrated that elevated PAC is an independent risk factor for incident CVD^11–15^. However, it is noteworthy that most published studies in this area have relied on a single measurement of PAC, neglecting the potential effects of heterogeneous long-term PAC patterns^16–19^. This limitation may bias the true relationship between PAC and CVD, favoring the null hypothesis. The formation of severe and complex intramural lesions leading to plaque rupture often takes years, despite the rapid manifestation of CVD^20,21^. Thus, longitudinal studies are essential for a comprehensive understanding of how long-term PAC trajectory patterns influence the development of CVD.

This study sought to ascertain the trajectories of PAC among 2254 hypertensive adults across a 6-year exposure period and investigate the relationship between these trajectories and the subsequent risk of CVD. We hypothesize that PAC trajectories can serve as predictors for the development of CVD. Therefore, examining patterns of change in PAC prior to the diagnosis of CVD may enhance the effectiveness of preventive and therapeutic interventions for this disease.

## Material and methods

### Study population

**Figure 1:**
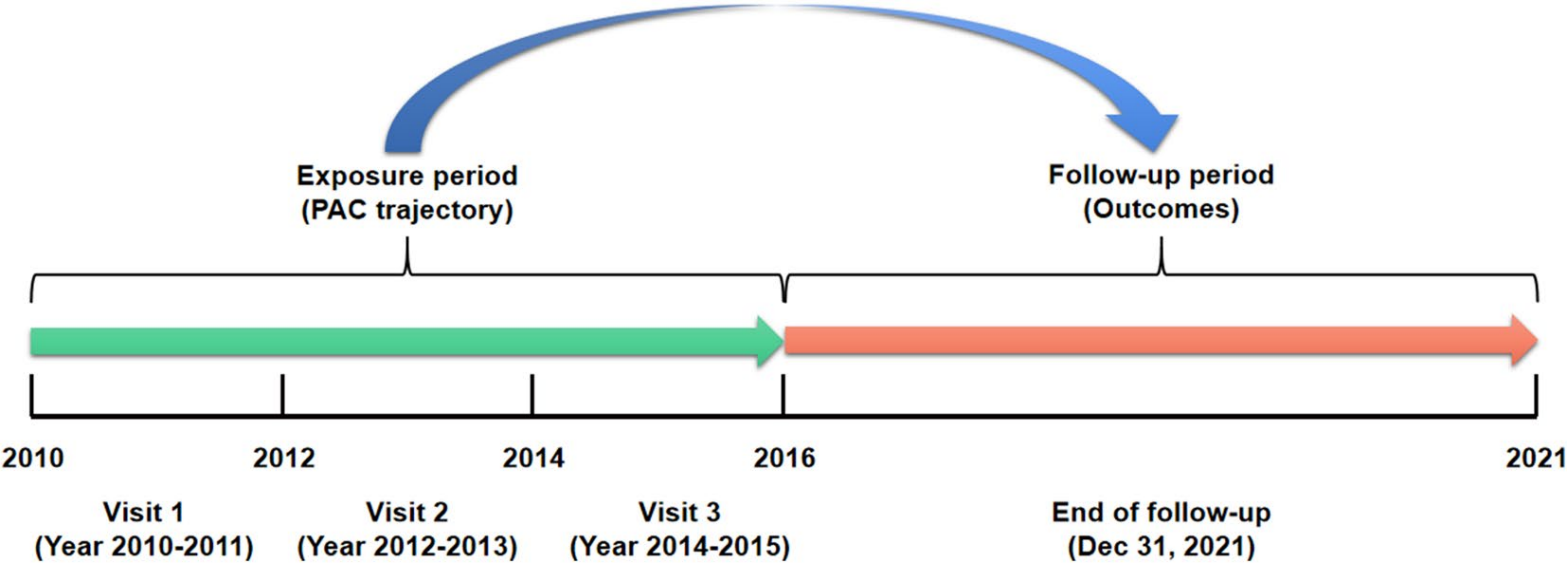
Description of the study's exposure and follow-up periods.

A cohort of 18,609 hypertensive adults was recruited from the People's Hospital of Xinjiang Uygur Autonomous Region and advised to undergo physical examinations at least once every 1 to 2 years. The initial three surveys (2010–2011, 2012–2013, and 2014–2015) enrolled 8179 participants. For the present analysis, 1124 participants were excluded due to a lack of follow-up data or less than 1 year of follow-up after 2016. Furthermore, we excluded 963 participants who had been diagnosed with myocardial infarction (MI), stroke, or cancer in or before 2016 (the baseline for this analysis). In addition, 3838 participants were excluded because PAC data from the three preceding surveys were unavailable. Therefore, a total of 2254 participants were ultimately included in the study (Fig. S1). PAC trajectory patterns were developed from 2010 to 2016 to predict CVD risk from 2016 to 2021 (Fig. 1). Ethical approval was secured from the Ethical Committee (No. KY2022080906), and the study adhered to the STROBE statement guidelines. We confirmed that all methods were performed in accordance with relevant guidelines and regulations, and all participants provided written informed consent.

### Covariate collection and definitions

Demographic, lifestyle, laboratory, medication, and medical history data were collected from electronic medical records for this study. Anthropometric data, including body mass index (BMI) and systolic and diastolic blood pressures (SBP and DBP), were collected using standardized methods. Alcohol use and smoking habits were categorized as either current or not. Blood samples were collected while the subjects were fasting. The central laboratory of the hospital measured the levels of high-density lipoprotein (HDL) cholesterol, low-density lipoprotein (LDL) cholesterol, fasting plasma glucose (FPG), total cholesterol (TC), triglycerides (TG), uric acid (UA), and high-sensitivity C-reactive protein (hs-CRP). PAC was measured using the radioimmunoassay method (DSL-8600 ACTIVE Aldosterone Coated Tube Radioimmunoassay Kit; Diagnostic Systems Laboratories, Webster, TX). Detailed descriptions of the measurement procedures can be found in previous studies^22,23^. The estimated glomerular filtration rate (eGFR) was calculated using the CKD-EPI formula^24^. A list of the medications used in the study can be found in Table S1. The medical history included records of hypertension, dyslipidemia, diabetes, chronic kidney disease (CKD), and primary aldosteronism (PA). For detailed information on the diagnostic criteria for the aforementioned medical history, please refer to the Supplemental Material. Comorbidities were characterized using the Charlson Comorbidity Index (CCI), as previously described^25^.

### Outcome measurement and follow-up

The primary endpoint of the study was the first occurrence of CVD, which encompasses both stroke and MI. Comprehensive definitions of CVD can be found in the Supplemental Materials. To obtain information regarding each participant's endpoint event, we gathered data from various sources, including hospital records, regional sickness and death registration systems, reconciliation with the national health insurance system, and interviews for confirmation. The follow-up period began in 2016 and ended with the earliest occurrence of CVD diagnosis, death, or on December 31, 2021, whichever occurred first.

### Statistical analysis

**Figure 2:**
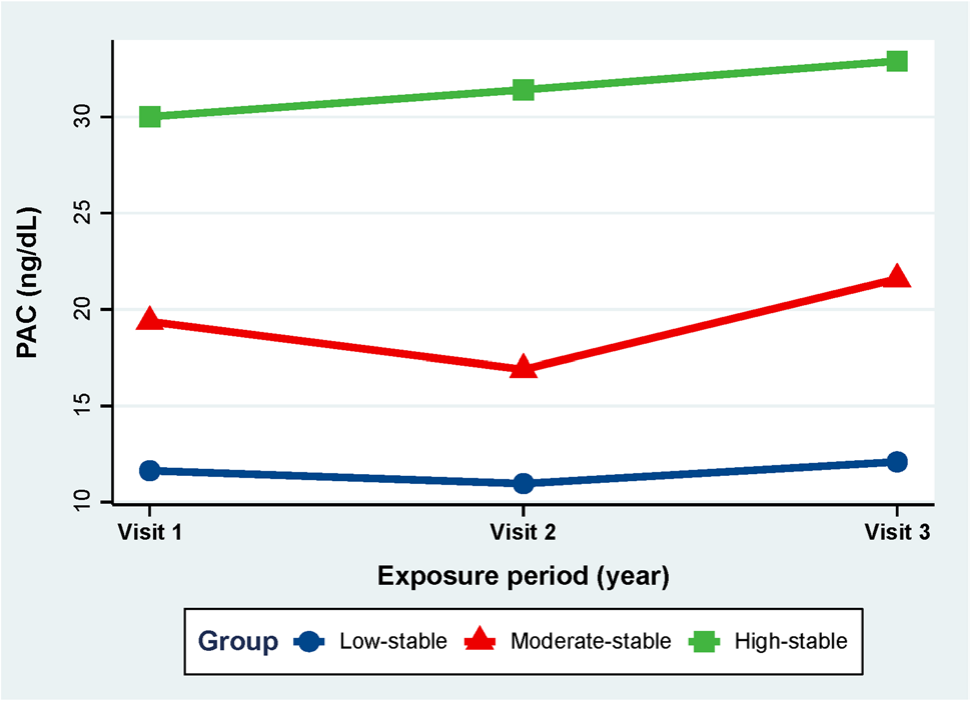
Trajectories of PAC.

Latent mixture modeling in the SAS Proc Traj was utilized to identify patterns of PAC trajectories ranging from 2010 to 2016^26^. The model with three classes was determined to have the best fit using the Bayesian information criterion (BIC), which was used to assess the model fit (Fig. 2 and Table S2). Missing covariate data (less than 10% missing) was imputed using 10 multiple imputations. To compare the disease-free survival rates between the groups, Kaplan–Meier and log-rank analyses were applied. The proportional hazards assumption was evaluated but not violated based on Schoenfeld residuals. Cox regression analyses were used to examine the relationship between PAC trajectory patterns and CVD risk. Hazard ratios (HR) and 95% confidence intervals (CI) were reported. Additionally, stratified analyses were performed, and interactions between subgroups were tested. Several sensitivity analyses were conducted to verify the reliability of the findings. More detailed information on statistical analysis is provided in the Supplemental Material. Any two-sided P value < 0.05 was statistically significant. Statistical analyses were performed using SAS 9.4 and R 4.1.1.

## Results

### Baseline characteristics

Table 1 provides a summary of the fundamental characteristics of 2254 hypertensive patients in 2016. These characteristics were based on the patterns of plasma aldosterone concentration (PAC) trajectories observed during the period of 2006–2010. Specifically, we identified three distinct patterns: low-stable (n = 1262), moderate-stable (n = 744), and high-stable (n = 248), as depicted in Fig. 2. Statistically significant differences were observed among the three groups in terms of heart rate, SBP, DBP, levels of TG, HDL cholesterol, FPG, UA, and BMI. Additionally, there were notable variations in the prevalence of diabetes, CKD, PA, and dyslipidemia, as well as differences in smoking status and the proportion of participants taking statins, aspirin, angiotensin-converting enzyme inhibitors (ACEIs)/angiotensin receptor blockers (ARBs), spironolactone, and glucose-lowering agents. However, no significant differences were observed among the three groups in terms of age, sex, TC, LDL cholesterol, eGFR, drinking status, or the proportion of participants taking beta-blockers and calcium channel blockers.

**Table 1:** Baseline characteristics of participants per trajectory of PAC.

Variables	Low-stable	Moderate-stable	High-stable	P-value
Subjects, n	1262	744	248	
Age, years	54.22 ± 9.52	53.66 ± 9.34	54.65 ± 10.00	0.271
Male, n (%)	726 (57.53%)	428 (57.53%)	131 (52.82%)	0.369
Heart rate, bpm	80.87 ± 10.90	82.34 ± 10.78	83.71 ± 10.92	< 0.001
DBP, mmHg	88.15 ± 14.59	92.31 ± 14.93	94.19 ± 14.53	< 0.001
SBP, mmHg	144.18 ± 20.27	147.34 ± 20.82	149.57 ± 22.86	< 0.001
BMI, kg/m^2^	24.97 ± 2.42	25.11 ± 2.30	26.00 ± 1.92	< 0.001
Current smoking, n (%)	384 (30.43%)	276 (37.10%)	97 (39.11%)	0.001
Current drinking, n (%)	407 (32.25%)	264 (35.48%)	93 (37.50%)	0.150
Laboratory parameters				
Total cholesterol, mmol/L	4.50 ± 0.97	4.54 ± 1.09	4.59 ± 1.04	0.414
Triglycerides, mmol/L	1.55 ± 0.83	2.18 ± 1.78	2.52 ± 1.80	< 0.001
HDL cholesterol, mmol/L	1.22 ± 0.28	0.91 ± 0.19	0.86 ± 0.17	< 0.001
LDL cholesterol, mmol/L	2.58 ± 0.78	2.67 ± 0.83	2.62 ± 0.80	0.064
FPG, mmol/L	5.07 ± 1.17	5.69 ± 2.43	6.07 ± 2.71	< 0.001
Hs-CRP, mg/L	1.59 (0.66–3.61)	1.63 (0.74–3.38)	1.78 (0.95–3.79)	0.083
Uric acid, μmol/L	302.47 ± 85.49	365.20 ± 94.83	381.29 ± 97.69	< 0.001
eGFR, ml/min/1.73 m^2^	97.48 ± 19.36	96.70 ± 19.61	98.71 ± 21.50	0.360
Medical history, n (%)				
Dyslipidemia	439 (34.79%)	341 (45.83%)	119 (47.98%)	< 0.001
Diabetes mellitus	146 (11.57%)	126 (16.94%)	51 (20.56%)	< 0.001
Chronic kidney disease	24 (1.90%)	41 (5.51%)	21 (8.47%)	< 0.001
Primary aldosteronism	34 (2.69%)	46 (6.18%)	48 (19.35%)	< 0.001
Charlson comorbidity index				< 0.001
0	852 (67.51%)	425 (57.12%)	131 (52.82%)	
1	219 (17.35%)	167 (22.45%)	61 (24.60%)	
≥ 2	191 (15.13%)	152 (20.43%)	56 (22.58%)	
Medications, n (%)				
Aspirins	486 (38.51%)	321 (43.15%)	114 (45.97%)	0.028
Statins	331 (26.23%)	234 (31.45%)	85 (34.27%)	0.006
ACEI/ARB	753 (59.67%)	502 (67.47%)	171 (68.95%)	< 0.001
Beta-blocker	405 (32.09%)	263 (35.35%)	86 (34.68%)	0.298
Calcium channel blockers	1022 (80.98%)	625 (84.01%)	210 (84.68%)	0.138
Spironolactone	60 (4.75%)	81 (10.89%)	40 (16.13%)	< 0.001
Insulin	34 (2.69%)	52 (6.99%)	22 (8.87%)	< 0.001
Oral antidiabetic drugs	73 (5.78%)	74 (9.95%)	30 (12.10%)	< 0.001

Data are presented as mean ± standard deviation, median (interquartile range), or N (%).

PAC, plasma aldosterone concentration; DBP, diastolic blood pressure; SBP, systolic blood pressure; BMI, body mass index; FPG, fasting plasma glucose; LDL, low-density lipoprotein; HDL, high-density lipoprotein; hs-CRP, high-sensitivity C-reactive protein; GFR, estimated glomerular filtration rate; ARB, angiotensin receptor blockers; ACEI, angiotensin-converting-enzyme inhibitors.

### Relationship between PAC trajectory patterns and CVD risk

**Figure 3:**
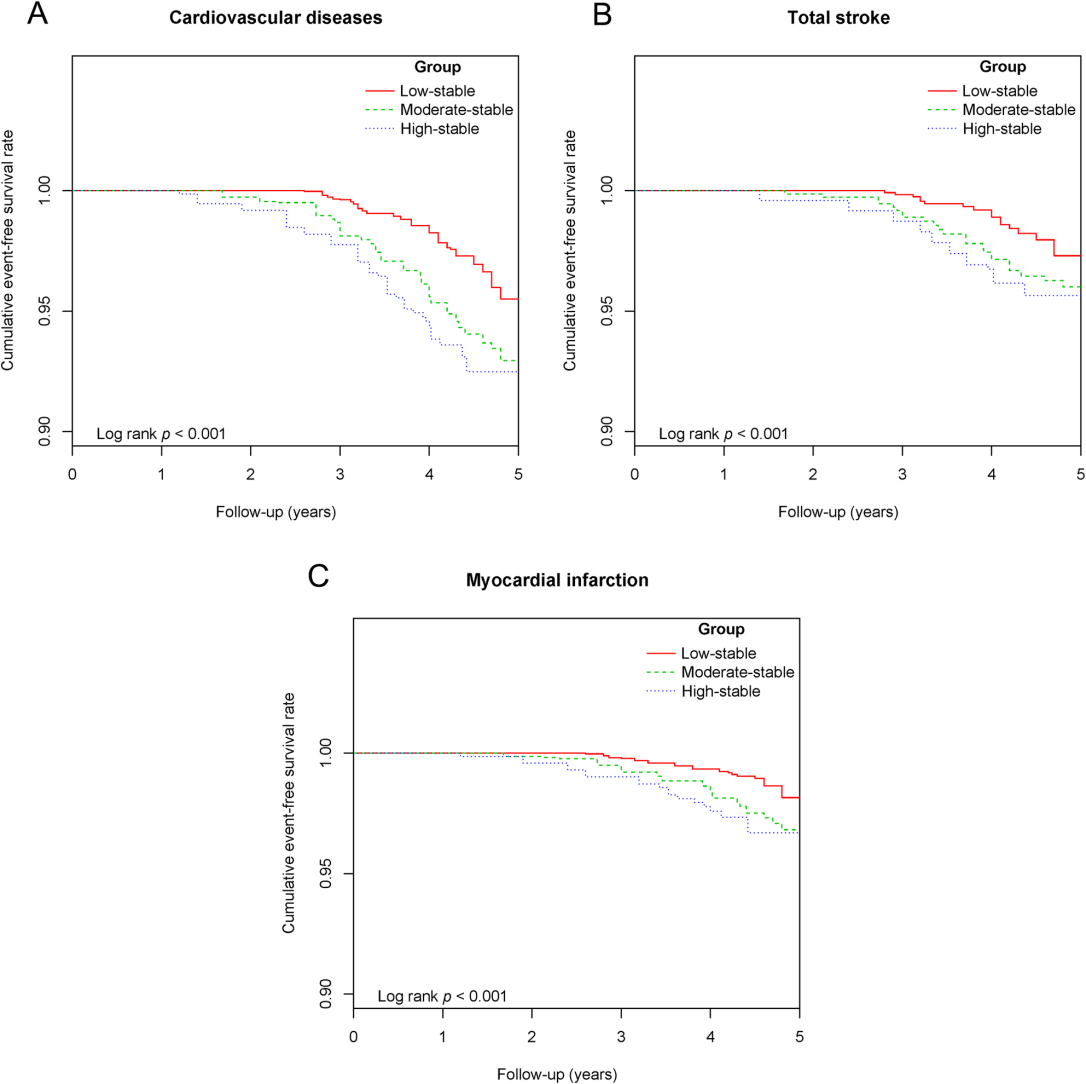
Kaplan–Meier estimation of cardiovascular disease by PAC trajectory patterns.

During a median follow-up period of 4.10 (3.37–4.50) years, we identified 82 cases of incident CVD, consisting of 33 cases of MI and 49 cases of stroke. The Kaplan–Meier curve demonstrated that participants in the high-stable PAC group exhibited a significantly elevated risk of CVD, stroke, and MI compared to other trajectory groups (log-rank P < 0.001 for all) (Fig. 3A–C). Among the three PAC models, the high-stability pattern was associated with the highest risk of CVD (Table 2). In model 4, the HRs were 2.19 (95% CI 1.59–3.11) for the moderate-stable pattern and 2.56 (95% CI 1.68–3.91) for the high-stable pattern as opposed to the low-stable pattern. The results for stroke and MI were consistent with those for CVD.

**Table 2:** Associations between PAC trajectory patterns and risk of CVD.

Exposure	Model 1	Model 2	Model 3	Model 4
Cardiovascular disease				
PAC trajectories				
Low-stable	1.00 (ref)	1.00 (ref)	1.00 (ref)	1.00 (ref)
Moderate-stable	2.11 (1.57, 2.84)	2.17 (1.61, 2.93)	2.19 (1.60, 3.01)	2.19 (1.59, 3.01)
High-stable	2.76 (1.91, 4.01)	2.81 (1.93, 4.09)	2.60 (1.70, 3.97)	2.56 (1.68, 3.91)
Total stroke				
PAC trajectories				
Low-stable	1.00 (ref)	1.00 (ref)	1.00 (ref)	1.00 (ref)
Moderate-stable	2.07 (1.42, 3.03)	2.14 (1.46, 3.15)	2.42 (1.62, 3.63)	2.36 (1.57, 3.56)
High-stable	2.60 (1.61, 4.20)	2.66 (1.64, 4.33)	2.90 (1.68, 5.01)	2.84 (1.64, 4.91)
Myocardial infarction				
PAC trajectories				
Low-stable	1.00 (ref)	1.00 (ref)	1.00 (ref)	1.00 (ref)
Moderate-stable	2.18 (1.36, 3.51)	2.22 (1.38, 3.59)	1.91 (1.15, 3.17)	1.97 (1.18, 3.28)
High-stable	3.03 (1.69, 5.45)	3.03 (1.67, 5.49)	2.30 (1.18, 4.47)	2.29 (1.17, 4.46)

Model 1: adjusted for age and sex.

Model 2: further adjusted for smoking status, alcohol consumption, history of diabetes, dyslipidemia, chronic kidney disease, primary aldosteronism, and CCI.

Model 3: further adjusted for DBP, SBP, BMI, UA, eGFR, TC, TG, HDL-C, LDL-C, FPG, and hs-CRP.

Model 4: further adjusted for the use of antihypertensive drugs, hypoglycemic drugs, statins, spironolactone, and aspirin.

### Subgroup and sensitivity analysis

The associations between patterns of PAC trajectories and CVD, stratified by age, sex, BMI, smoking status, drinking status, and diabetes, are presented in Table 3. No significant interactions were found between the PAC trajectory patterns and age, sex, BMI, smoking or drinking status, or diabetes (P for interaction > 0.05 for all). The results did not change substantially in the sensitivity analysis by excluding subjects diagnosed with CVD in the two years before follow-up, individuals with a history of PA, or participants with CCI ≥ 2 (Tables  S3–S5). Moreover, analyses without multiple imputations of covariates produced results that aligned with those of the primary analysis (Table  S6). Accounting for the competing risk of mortality in an additional analysis yielded outcomes comparable to those in the primary analysis (Table S7). Furthermore, we observed that significant correlations between PAC trajectories and CVD risk continued to persist, even after further adjusting for the baseline level of PAC (Table S8). Lastly, the E-values computed for the study's outcomes ranged from 3.35 to 5.13, indicating a moderate strength of evidence against potential unmeasured confounding variables (Table S9).

**Table 3:** Subgroup analyses for the association between PAC trajectory patterns and CVD risk.

Stratification	PAC trajectories			P for interaction
	Low-stable	Moderate-stable	High-stable	
Sex				0.341
Female	1.00 (ref)	2.42 (1.44, 4.07)	1.89 (0.82, 4.35)	
Male	1.00 (ref)	1.78 (1.20, 2.65)	2.51 (1.59, 3.97)	
Age				0.118
< 60 years	1.00 (ref)	2.29 (1.60, 3.29)	1.85 (1.10, 3.12)	
≥ 60 years	1.00 (ref)	1.54 (0.80, 2.96)	3.38 (1.62, 7.08)	
BMI				0.656
< 25 kg/m^2^	1.00 (ref)	2.20 (1.39, 3.47)	2.04 (1.07, 3.91)	
≥ 25 kg/m^2^	1.00 (ref)	1.86 (1.20, 2.89)	2.36 (1.36, 4.10)	
Smoking status				0.486
Not current	1.00 (ref)	2.37 (1.64, 3.41)	2.26 (1.36, 3.76)	
Current	1.00 (ref)	1.67 (0.91, 3.08)	2.40 (1.16, 4.98)	
Drinking status				0.230
Not current	1.00 (ref)	2.02 (1.41, 2.90)	1.89 (1.13, 3.17)	
Current	1.00 (ref)	1.80 (0.95, 3.42)	3.14 (1.51, 6.51)	

HRs were obtained after adjustment for the same variables as model 4 in Table 2, except for the stratified variable.

## Discussion

This study represents the first longitudinal investigation, to our knowledge, that comprehensively examines the effect of observed changes in PAC on the risk of CVD. In our investigation, we categorized PAC trajectory patterns between 2010 and 2016 into three distinct groups: low-stable, moderate-stable, and high-stable. We found that hypertensive patients with a persistently high PAC level over the long term had a significantly elevated risk of CVD during the follow-up period, compared to those in the low-stable group. Importantly, these findings were consistent across various sensitivity and subgroup analyses, reinforcing their robustness. These results suggest that monitoring changes in PAC trajectory could serve as a valuable approach for identifying individuals at risk of CVD and aiding in the prevention of new-onset CVD in hypertensive patients.

Several observational studies in patients with heart failure have shown that PAC levels are closely linked to an elevated risk of recurrent myocardial infarction, severe heart failure, or cardiovascular mortality^16,18,19,27^. In a cohort of patients scheduled for coronary angiography, variation in PAC levels within the normal range was associated with increased all-cause and CVD mortality, independent of other major CVD risk factors^28^. Similarly, Hillaert et al. demonstrated that in patients with stable coronary artery disease, PAC levels were independently linked with the risk of major vascular events, vascular death, and atherosclerotic burden^29^. Ivanes et al. also found a strong independent association between PAC levels and the occurrence of acute ischemic events in patients with coronary artery disease without heart failure or acute myocardial infarction^16^. Notably, they observed a stronger association between higher PAC levels and overall CVD mortality and sudden cardiac death in patients with lower renal function^14^. A community-based cohort study conducted in the United States revealed a nonlinear relationship between higher PAC levels and increased CVD risk, independent of known cardiovascular risk factors. The use of renin–angiotensin–aldosterone system antagonists does not affect the relationship between PAC and CVD^11^. However, it is worth noting that a limited number of studies have reported a nonsignificant relationship between PAC and CVD. For instance, the KORA F4 study found a nonsignificant trend suggesting a positive association between higher PAC levels and cardiovascular mortality, stroke, and composite cardiovascular endpoints, but not with MI^30^. Another cohort study of patients with chronic renal insufficiency showed that higher PAC levels were independently associated with the development of congestive heart failure but not with end-stage renal disease, atherosclerotic events, or death^13^. These conflicting findings may be attributed to the fact that previous studies primarily focused on absolute PAC levels at baseline and subsequent CVD risk rather than considering changes in PAC over time. To address this gap in knowledge, our study adopted a longitudinal cohort design to explore the association between PAC trajectory patterns and CVD. By examining the relationship between PAC trajectory patterns and cardiovascular health status, our study contributes to the limited available evidence and introduces a potential novel indicator for the prevention of CVD.

The mechanisms linking a high-stable PAC trajectory to CVD risk are not completely understood. Several plausible mechanisms may contribute to this observation. Through a number of mechanisms, including the suppression of endothelial function, persistent intravascular fluid retention, and the production of target-organ inflammation and fibrosis, aldosterone probably aids in the development and/or progression of CVD^31,32^. First, the traditional action of excessive aldosterone causing incorrect volume retention directly causes increases in cardiac output, BP, glomerular filtration rate, and intracardiac volume^33,34^. Elevated BP, stemming from increased intravascular volume, raises the risk of heart disease, stroke, and CKD^35–38^. Chronic increases in left atrial volume can predispose to arrhythmias, particularly atrial fibrillation, whereas chronic increases in intracardiac volume are likely to raise the chance of developing heart failure, particularly in the presence of uncontrolled hypertension^39–42^. Renal plasma blood flow increases along with an increase in circulating volume. Increased glomerular dysfunction, such as proteinuria, is brought on by the corresponding rise in intraglomerular pressure and glomerular filtration rate^43–45^. Additionally, aldosterone may exert other effects on the pathophysiology of CVD, including atherogenesis, heightened sympathetic activity, impaired pressure reflex function, and increased thrombosis^31,46,47^.

To our knowledge, this is the first study to investigate the impact of PAC trajectory patterns on CVD risk in Chinese hypertensive patients. The strengths of this study encompass its longitudinal design, large sample size, repeated measurements of study variables, and robustness of the observed associations. Nevertheless, certain limitations should be acknowledged. Firstly, due to the observational nature of the study, causal conclusions cannot be drawn. Secondly, despite controlling for a range of potential confounders, we cannot completely exclude all residual and unmeasured confounders, such as genetic predisposition, diet, medication, and family history. Thirdly, the focus of data collection on stroke and MI may underestimate the overall incidence of CVD. Fourthly, this study population included only Chinese hypertensive patients and therefore should be extended to other population groups with caution. Fifthly, only three different stable trajectories were found in the current study, and further studies of other populations with less stable trajectories are required to investigate the role of PAC changes in the development of CVD. Lastly, multiple PAC measurements may be needed in clinical practice before determining the trajectory of patients, making their use limited in daily clinical practice.

## Conclusion

In summary, long-term PAC patterns were linked to altered CVD risk, and higher levels of PAC over time were significantly associated with an elevated risk of CVD in patients with hypertension. These findings underscore the potential of monitoring long-term PAC patterns as a valuable tool for identifying individuals at heightened risk of CVD. Nevertheless, additional studies need to be conducted to validate these findings.

### Supplementary Information

Supplementary Information.

## Data Availability

The manuscript contains all the evidence that supports the findings. The datasets used and/or analyzed in this study are available from the corresponding author on request if necessary.
