# Author Correction: Association of the trajectory of plasma aldosterone concentration with the risk of cardiovascular disease in patients with hypertension: a cohort study

**DOI:** 10.1038/s41598-024-60563-z

**Published:** 2024-04-29

**Authors:** Xintian Cai, Shuaiwei Song, Junli Hu, Qing Zhu, Di Shen, Wenbo Yang, Huimin Ma, Qin Luo, Jing Hong, Delian Zhang, Nanfang Li

**Affiliations:** Hypertension Center of People’s Hospital of Xinjiang Uygur Autonomous Region, Xinjiang Hypertension Institute, NHC Key Laboratory of Hypertension Clinical Research, Key Laboratory of Xinjiang Uygur Autonomous Region “Hypertension Research Laboratory”, Xinjiang Clinical Medical Research Center for Hypertension (Cardio-Cerebrovascular) Diseases, No. 91 Tianchi Road, Ürümqi, 830001 Xinjiang China

## Introduction

Correction to: *Scientific Reports* 10.1038/s41598-024-54971-4, published online 28 February 2024

The original version of this Article contained an error in the Author Information section, where an Equal Contribution statement was incorrectly included.

“These authors contributed equally: Xintian Cai and Shuaiwei Song.”

The original Article has been corrected.

