# Supplementary material for: Association of the trajectory of plasma aldosterone concentration with the risk of cardiovascular disease in patients with hypertension: a cohort study
Source: Sci Rep. 2024 Feb 28;14:4906. doi: 10.1038/s41598-024-54971-4 (PMC10902285; doi:10.1038/s41598-024-54971-4)
Supplement: Supplementary file 1 — Supplementary Information. [file 41598_2024_54971_MOESM1_ESM.docx]

Supplementary Material

# Supplemental material and methods

**Definitions of medical history**

Hypertension was defined by blood pressure (BP) ≥140/90 or International Classification of Diseases -10 (ICD-10) codes I10-I13, I15, or the use of antihypertensive medication. Diabetes mellitus was defined by fasting plasma glucose (FPG) ≥ 7.0 mmol/L, random plasma glucose ≥11.1 mmol/L, 2-h glucose ≥11.1 mmol/L after oral glucose tolerance test, or disease codes of E10-E14, as well as the use of hypoglycemic medication. According to the guideline in China, dyslipidemia was defined as total cholesterol (TC) ≥ 6.2 mmol/L, triglycerides (TG) ≥ 2.3 mmol/L, high-density lipoprotein (HDL) cholesterol < 1.0 mmol/L, low-density lipoprotein (LDL) cholesterol ≥ 4.1 mmol/L, or a code of E78, and use of lipid-lowering medication ^[1]^. Chronic kidney disease (CKD) was defined as an eGFR of <60 ml/min/1.73 m^2^ or disease code N18.

**Screening and diagnosis of primary aldosteronism (PA)**

Fasting blood samples were drawn between local time 09:00 am (Beijing time 11:00 am) after patients had seated for 30 minutes. All hormonal tests were performed as recommended by current guidelines ^[2]^. Patients were requested to stop taking antihypertensive agents, including diuretics, mineralocorticoid receptor antagonists, and beta-blockers, for 4–6 weeks before hormone testing. For patients with BP ≥ 160/100 mmHg, we switched their antihypertensive agents to alpha-blockers and/or calcium channel blockers (verapamil) for 4–6 weeks before hormone testing. In the presence of severe or symptomatic hypertension, the workup was made under antihypertensive medications known to affect measurements of plasma aldosterone concentration (PAC) and plasma renin activity (PRA). In addition, if patients suffered from hypokalemia (<3.5 mmol/L), serum K was corrected with oral K supplements as close as possible to 4.0 mmol/L, and all patients were recommended to maintain a full diet with dietary salt intake. PAC was measured using radioimmunoassay (DSL-8600 ACTIVE® Aldosterone Coated Tube Radioimmunoassay Kit; Diagnostic Systems Laboratories, Webster, TX, USA). PRA was measured by radioimmunoassay using commercial kits (Center of Beifang Biology Technique, Beijing, China). Plasma samples were divided into two parts: one for the determination of plasma angiotensin I (AIa) concentrations after reacting the sample with direct antibodies, and the other for the determination of plasma AIb concentrations following a 1-h incubation at 37 °C and then reacting the sample with direct antibodies. PRA was calculated using the following formula: [AIb-AIa]/h.

Screening and diagnosis data for PA were re-evaluated according to the 2016 Endocrine Society Guidelines ^[2]^ as in our recent studies ^[3]^. A positive screening test was defined as PRA < 1 ng/mL per hour and PAC ≥ 12 ng/dL, or aldosterone to renin ratio (ARR) ≥ 20. To avoid factitious inflation of ARR, PRA values were fixed at 0.20 ng/mL per hour, when PRA was <0.20 ng/mL per hour. In patients with a positive screening test, the salt infusion test (SIT) was performed as a confirmatory test. PA was defined if post-SIT PAC was ≥10 ng/dL or if hypokalemia was present and screening PAC was ≥20 ng/dL when without SIT, in those with positive screening tests ^[2]^.

**Definitions of CVD and follow-up**

The cardiovascular events included myocardial infarction (MI) or any stroke (hemorrhagic or ischemic stroke). MI, including ST-segment elevation myocardial infarction and non-ST-segment elevation myocardial infarction, was diagnosed based on the onset of angina pectoris, ischemic features in the electrocardiogram (ECG), and an increase in serum biomarkers such as cardiac troponin T, cardiac troponin I, or creatine kinase MB ^[4]^. Stroke was diagnosed based on neurological signs, clinical symptoms, and neuroimaging tests, including computed tomographic or magnetic resonance imaging, in alignment with the World Health Organization criteria ^[5]^. The follow-up period began in 2016 and ended with the earliest occurrence of CVD diagnosis, death, or on December 31, 2021, whichever occurred first. The follow-ups were performed by trained physicians who were blinded to the baseline data. To obtain information regarding each participant's endpoint event, we gathered data from various sources, including hospital records, regional sickness and death registration systems, reconciliation with the national health insurance system, and interviews for confirmation. All suspected cardiovascular events and causes of death were adjudicated by an independent clinical endpoint committee.

**Details of the statistical analyses**.

Trajectories of PAC were identified using group-based trajectory modeling with SAS PROC TRAJ ^[6]^. This method can automatically divide the study population into classes in such a way that participants in the same class tend to have similar trajectories of PAC change. We used a censored normal model appropriate for continuous outcomes. Model fit was assessed using the Bayesian information criterion (BIC). Initially, all PAC trajectories started with quadratic shapes and compared the BIC with the models of two, three, four, and five classes. The results indicated that the optimal number of trajectories was three (Figure 2). Next, we compared the model with different functional forms. Cubic, quadratic, and linear terms were considered and evaluated based on their significance level, starting with the highest polynomial. The optimal number of trajectories and trajectory shapes were determined by the following criteria: (1) improvement in the BIC score, (2) no less than 5% membership in each trajectory group, and (3) high group average posterior probabilities (> 0.7). In our final model, we had one pattern with a linear order term and two patterns with up to quadratic order terms (Table S2).

We imputed missing data for covariates ( < 10%) with 10 multiple imputations and chain equations using all covariates. Multiple imputations of missing data were used in all multivariable models to reduce the potential for bias due to missing data and improve efficiency by taking full advantage of our data. Estimates and their variances from the multiple imputation results were combined according to the Rubin method. Baseline characteristics were analyzed using descriptive statistics. Categorical variables were described as frequency and percentage. Continuous variables were described as mean (± standard deviation [SD]) for normally distributed data and as geometric mean and 95% confidential interval (CI) for data not normally distributed. Continuous variables were compared using one-way ANOVA, while categorical variables were compared using the chi-square test.

The person-years were determined from the date when the message was collected at baseline to either the date of CVD diagnosis, death, or the end of follow-up (the date of the last visit), whichever happened first. The Kaplan-Meier method was used to compute the cumulative incidence of CVD and subgroups of CVD. Differences between Kaplan-Meier curves were compared using the log-rank test. Schoenfeld residuals were generated to confirm the risk proportionality assumptions. The proportional hazards assumption was met for all Cox and competing risk models. Cox regression analysis was used to examine the association between PAC trajectories and the risk of CVD, stroke, and MI. Hazard ratios (HR) with 95% confidence intervals (CI) were reported. Four models were constructed. Model 1 was adjusted for age and sex. Model 2 was further adjusted for smoking status, alcohol consumption, history of diabetes, dyslipidemia, chronic kidney disease, primary aldosteronism, and CCI. Model 3 was additionally adjusted for DBP, SBP, BMI, UA, eGFR, TC, TG, HDL-C, LDL-C, FPG, and hs-CRP. Further adjustments were made for the use of antihypertensive drugs, hypoglycemic drugs, statins, spironolactone, and aspirin in Model 4. Stratified analyses were performed according to age (< 60 vs. ≥ 60 years), sex (female vs. male), smoking status (not current vs. current), alcohol drinking status (not current vs. current), and obesity status (BMI < 25 kg/m^2^ vs. ≥ 25 kg/m^2^). P values for interactions were evaluated using interaction terms and likelihood ratio tests.

Multiple sensitivity analyses were presented here to examine the robustness of our findings. First, to minimize the effect of reverse causality, we performed additional analyses by excluding participants diagnosed with CVD during the first 2 years of follow-up. Second, a sensitivity analysis excluding individuals with a history of primary aldosteronism was also performed. Third, considering that comorbidities may have an additional effect on CVD, we excluded participants with CCI ≥2. Fourth, we repeated our analyses without using multiple imputations. Fifth, we conducted the Fine-Grey subdistribution hazard model to account for death as a competing risk. Sixth, to control the impact of baseline PAC, we performed an additional sensitivity analysis with covariates, including the baseline PAC in the models. Lastly, the E-value was computed to estimate the effect of unobserved confounding (5).

P values were two-sided, and statistical significance was set at P < 0.05. Statistical analyses were done using SAS version 9.4 and R version 4.1.1.

# Supplementary Figures and Tables

## Supplementary Tables

**Table S1**. List of medications included in the study.

| Drug class | Drug name |
| --- | --- |
| Aspirin | Aspirin |
| Beta-blocker | Atenolol, bisoprolol, carvedilol, metoprolol, propranolol |
| Angiotensin-converting enzyme inhibitors or angiotensin receptor blockers | Azilsartan, candestartan, captopril, enalapril, fosinopril, irbesartan, losartan, olmesartan, ramipril telmisartan, valsartan |
| Calcium channel blockers | Amlodipine, diltiazem, felodipine, lercanidipine, nifedipine,  verapamil |
| Spironolactone | Spironolactone |
| Statin | Atorvastatin, fluvastatin, pitavastatin, rosuvastatin, simvastatin |
| Oral antidiabetic agents | Metformin, glipizide, gliclazide, glimepiride, glyburide, alogliptin, linagliptin, sitagliptin, vidagliptin, saxagliptin, acarbose, nateglinide, meglitinide, repaglinde, pioglitzone,dulaglutide, exenatide, liraglutide |
| Insulin | Rapid, short, intermediate and long-acting insulins |

**Table S2**. Fit statistics for PAC group trajectories.

| Fit statistic | Group | | | |
| --- | --- | --- | --- | --- |
|  | 2 | **3** | 4 | 5 |
| AIC* | -94544.3868 | **-91230.43299** | -91678.56202 | -91348.29141 |
| BIC* | -94629.35167 | **-91363.94922** | -91860.67006 | -91578.95081 |
| Average posterior probability | 0.91/0.91 | **0.87/0.90/0.87** | 0.84/0.86/0.86/0.84 | 0.81/0.83/0.85/0.83/0.81 |
| Group percent | Group1, 62.0% | **Group1, 56.0%** | Group1, 16.1% | Group1, 4.1% |
|  | Group2, 38.0% | **Group2, 33.0%** | Group2, 31.9% | Group2, 19.9% |
|  |  | **Group3, 11.0%** | Group3, 37.9% | Group3, 38.6% |
|  |  |  | Group4, 14.1% | Group4, 27.9% |
|  |  |  |  | Group5, 9.5% |

AIC: Akaike information criterion; BIC: Bayesian information criteria.

*A lower absolute value suggests a better model fit.

**Table S3.** Sensitivity analysis excluded outcome events within the first 2 years of follow-up.

| Exposure | Model 1 | Model 2 | Model 3 | Model 4 |
| --- | --- | --- | --- | --- |
| **Cardiovascular disease** |  |  |  |  |
| PAC trajectories |  |  |  |  |
| Low-stable | 1.00 (ref) | 1.00 (ref) | 1.00 (ref) | 1.00 (ref) |
| Moderate-stable | 2.71 (1.99, 3.70) | 2.86 (2.09, 3.91) | 3.59 (2.55, 5.05) | 3.64 (2.57, 5.17) |
| High-stable | 2.82 (1.93, 4.12) | 2.94 (2.00, 4.32) | 2.89 (1.87, 4.46) | 2.89 (1.86, 4.50) |
| **Total stroke** |  |  |  |  |
| PAC trajectories |  |  |  |  |
| Low-stable | 1.00 (ref) | 1.00 (ref) | 1.00 (ref) | 1.00 (ref) |
| Moderate-stable | 2.60 (1.75, 3.85) | 2.76 (1.85, 4.10) | 3.85 (2.49, 5.95) | 3.83 (2.46, 5.97) |
| High-stable | 2.59 (1.59, 4.22) | 2.73 (1.67, 4.48) | 3.05 (1.75, 5.34) | 3.07 (1.74, 5.41) |
| **Myocardial infarction** |  |  |  |  |
| PAC trajectories |  |  |  |  |
| Low-stable | 1.00 (ref) | 1.00 (ref) | 1.00 (ref) | 1.00 (ref) |
| Moderate-stable | 2.91 (1.76, 4.83) | 3.03 (1.82, 5.07) | 3.42 (1.95, 5.99) | 3.72 (2.10, 6.59) |
| High-stable | 3.22 (1.76, 5.91) | 3.28 (1.77, 6.07) | 2.84 (1.42, 5.68) | 2.80 (1.38, 5.66) |

Model 1: adjusted for age and sex

Model 2: further adjusted for smoking status, alcohol consumption, history of diabetes, dyslipidemia, chronic kidney disease, primary aldosteronism, and CCI

Model 3: further adjusted for DBP, SBP, BMI, UA, eGFR, TC, TG, HDL-C, LDL-C, FPG, and hs-CRP

Model 4: further adjusted for the use of antihypertensive drugs, hypoglycemic drugs, statins, spironolactone, and aspirin

**Table S4**. Sensitivity analysis of excluding individuals with prevalent primary aldosteronism at baseline.

| Exposure | Model 1 | Model 2 | Model 3 | Model 4 |
| --- | --- | --- | --- | --- |
| **Cardiovascular disease** |  |  |  |  |
| PAC trajectories |  |  |  |  |
| Low-stable | 1.00 (ref) | 1.00 (ref) | 1.00 (ref) | 1.00 (ref) |
| Moderate-stable | 2.13 (1.57, 2.88) | 2.19 (1.62, 2.97) | 2.17 (1.57, 2.99) | 2.15 (1.56, 2.98) |
| High-stable | 2.42 (1.62, 3.60) | 2.43 (1.62, 3.64) | 2.28 (1.46, 3.55) | 2.24 (1.43, 3.49) |
| **Total stroke** |  |  |  |  |
| PAC trajectories |  |  |  |  |
| Low-stable | 1.00 (ref) | 1.00 (ref) | 1.00 (ref) | 1.00 (ref) |
| Moderate-stable | 2.09 (1.42, 3.10) | 2.17 (1.47, 3.23) | 2.38 (1.57, 3.62) | 2.29 (1.50, 3.49) |
| High-stable | 2.19 (1.29, 3.71) | 2.21 (1.29, 3.78) | 2.54 (1.43, 4.52) | 2.44 (1.37, 4.35) |
| **Myocardial infarction** |  |  |  |  |
| PAC trajectories |  |  |  |  |
| Low-stable | 1.00 (ref) | 1.00 (ref) | 1.00 (ref) | 1.00 (ref) |
| Moderate-stable | 2.18 (1.36, 3.51) | 2.22 (1.38, 3.59) | 1.93 (1.16, 3.22) | 2.00 (1.20, 3.35) |
| High-stable | 2.78 (1.51, 5.12) | 2.76 (1.48, 5.12) | 2.03 (1.02, 4.06) | 2.02 (1.01, 4.05) |

Model 1: adjusted for age and sex

Model 2: further adjusted for smoking status, alcohol consumption, history of diabetes, dyslipidemia, and chronic kidney disease

Model 3: further adjusted for DBP, SBP, BMI, UA, eGFR, TC, TG, HDL-C, LDL-C, FPG, and hs-CRP

Model 4: further adjusted for the use of antihypertensive drugs, hypoglycemic drugs, statins, spironolactone, and aspirin

**Table S5**. Sensitivity analysis excluding subjects with CCI ≥2 at baseline.

| Exposure | Model 1 | Model 2 | Model 3 | Model 4 |
| --- | --- | --- | --- | --- |
| **Cardiovascular disease** |  |  |  |  |
| PAC trajectories |  |  |  |  |
| Low-stable | 1.00 (ref) | 1.00 (ref) | 1.00 (ref) | 1.00 (ref) |
| Moderate-stable | 2.32 (1.66, 3.25) | 2.45 (1.75, 3.43) | 2.43 (1.70, 3.46) | 2.41 (1.69, 3.45) |
| High-stable | 3.01 (1.96, 4.61) | 3.14 (2.04, 4.84) | 2.95 (1.81, 4.83) | 2.86 (1.75, 4.68) |
| **Total stroke** |  |  |  |  |
| PAC trajectories |  |  |  |  |
| Low-stable | 1.00 (ref) | 1.00 (ref) | 1.00 (ref) | 1.00 (ref) |
| Moderate-stable | 2.32 (1.50, 3.57) | 2.44 (1.58, 3.77) | 2.69 (1.70, 4.24) | 2.63 (1.66, 4.18) |
| High-stable | 3.00 (1.73, 5.18) | 3.12 (1.79, 5.43) | 3.26 (1.74, 6.11) | 3.02 (1.61, 5.66) |
| **Myocardial infarction** |  |  |  |  |
| PAC trajectories |  |  |  |  |
| Low-stable | 1.00 (ref) | 1.00 (ref) | 1.00 (ref) | 1.00 (ref) |
| Moderate-stable | 2.33 (1.37, 3.96) | 2.47 (1.45, 4.20) | 2.10 (1.19, 3.69) | 2.15 (1.22, 3.79) |
| High-stable | 3.03 (1.54, 5.96) | 3.15 (1.59, 6.27) | 2.58 (1.18, 5.65) | 2.64 (1.20, 5.82) |

Model 1: adjusted for age and sex

Model 2: further adjusted for smoking status, alcohol consumption, history of diabetes, dyslipidemia, chronic kidney disease, and primary aldosteronism

Model 3: further adjusted for DBP, SBP, BMI, UA, eGFR, TC, TG, HDL-C, LDL-C, FPG, and hs-CRP

Model 4: further adjusted for the use of antihypertensive drugs, hypoglycemic drugs, statins, spironolactone, and aspirin

**Table S6**. Sensitivity analysis with the raw datasets (without imputation for missing values).

| Exposure | Model 1 | Model 2 | Model 3 | Model 4 |
| --- | --- | --- | --- | --- |
| **Cardiovascular disease** |  |  |  |  |
| PAC trajectories |  |  |  |  |
| Low-stable | 1.00 (ref) | 1.00 (ref) | 1.00 (ref) | 1.00 (ref) |
| Moderate-stable | 2.44 (1.76, 3.39) | 2.57 (1.85, 3.58) | 2.54 (1.79, 3.62) | 2.56 (1.79, 3.65) |
| High-stable | 3.39 (2.26, 5.08) | 3.53 (2.34, 5.31) | 2.89 (1.82, 4.59) | 2.81 (1.77, 4.46) |
| **Total stroke** |  |  |  |  |
| PAC trajectories |  |  |  |  |
| Low-stable | 1.00 (ref) | 1.00 (ref) | 1.00 (ref) | 1.00 (ref) |
| Moderate-stable | 2.52 (1.63, 3.92) | 2.73 (1.76, 4.26) | 3.02 (1.88, 4.84) | 2.96 (1.84, 4.78) |
| High-stable | 3.57 (2.09, 6.09) | 3.84 (2.24, 6.59) | 3.65 (1.98, 6.75) | 3.41 (1.85, 6.28) |
| **Myocardial infarction** |  |  |  |  |
| PAC trajectories |  |  |  |  |
| Low-stable | 1.00 (ref) | 1.00 (ref) | 1.00 (ref) | 1.00 (ref) |
| Moderate-stable | 2.34 (1.43, 3.83) | 2.37 (1.44, 3.89) | 2.06 (1.21, 3.51) | 2.16 (1.26, 3.68) |
| High-stable | 3.16 (1.70, 5.87) | 3.13 (1.67, 5.87) | 2.19 (1.08, 4.44) | 2.15 (1.05, 4.39) |

Model 1: adjusted for age and sex

Model 2: further adjusted for smoking status, alcohol consumption, history of diabetes, dyslipidemia, chronic kidney disease, primary aldosteronism, and CCI

Model 3: further adjusted for DBP, SBP, BMI, UA, eGFR, TC, TG, HDL-C, LDL-C, FPG, and hs-CRP

Model 4: further adjusted for the use of antihypertensive drugs, hypoglycemic drugs, statins, spironolactone, and aspirin

**Table S7**. Sensitivity analysis was conducted using the Fine-Gray competing risk model, considering non-CVD deaths as competing risk events.

| Exposure | Model 1 | Model 2 | Model 3 | Model 4 |
| --- | --- | --- | --- | --- |
| **Cardiovascular disease** |  |  |  |  |
| PAC trajectories |  |  |  |  |
| Low-stable | 1.00 (ref) | 1.00 (ref) | 1.00 (ref) | 1.00 (ref) |
| Moderate-stable | 2.14 (1.59, 2.89) | 2.12 (1.57, 2.87) | 2.12 (1.54, 2.91) | 2.12 (1.54, 2.92) |
| High-stable | 2.87 (1.98, 4.15) | 2.81 (1.93, 4.10) | 2.57 (1.68, 3.92) | 2.54 (1.66, 3.87) |
| **Total stroke** |  |  |  |  |
| PAC trajectories |  |  |  |  |
| Low-stable | 1.00 (ref) | 1.00 (ref) | 1.00 (ref) | 1.00 (ref) |
| Moderate-stable | 2.14 (1.46, 3.13) | 2.12 (1.44, 3.12) | 2.40 (1.60, 3.60) | 2.33 (1.55, 3.52) |
| High-stable | 2.71 (1.68, 4.38) | 2.68 (1.65, 4.36) | 2.94 (1.70, 5.09) | 2.88 (1.66, 4.98) |
| **Myocardial infarction** |  |  |  |  |
| PAC trajectories |  |  |  |  |
| Low-stable | 1.00 (ref) | 1.00 (ref) | 1.00 (ref) | 1.00 (ref) |
| Moderate-stable | 2.16 (1.34, 3.49) | 2.13 (1.31, 3.45) | 1.78 (1.06, 2.97) | 1.85 (1.10, 3.10) |
| High-stable | 3.12 (1.73, 5.60) | 3.01 (1.66, 5.46) | 2.18 (1.12, 4.25) | 2.18 (1.12, 4.25) |

Competing risk regression: sub-hazard ratios with the corresponding 95% confidence intervals are shown.

Model 1: adjusted for age and sex

Model 2: further adjusted for smoking status, alcohol consumption, history of diabetes, dyslipidemia, chronic kidney disease, primary aldosteronism, and CCI

Model 3: further adjusted for DBP, SBP, BMI, UA, eGFR, TC, TG, HDL-C, LDL-C, FPG, and hs-CRP

Model 4: further adjusted for the use of antihypertensive drugs, hypoglycemic drugs, statins, spironolactone, and aspirin

**Table S8**. Sensitivity analysis of additionally adjusted for the baseline PAC.

| Exposure | Model 5 |
| --- | --- |
| **Cardiovascular disease** |  |
| PAC trajectories |  |
| Low-stable | 1.00 (ref) |
| Moderate-stable | 2.18 (1.59, 3.00) |
| High-stable | 2.48 (1.62, 3.80) |
| **Total stroke** |  |
| PAC trajectories |  |
| Low-stable | 1.00 (ref) |
| Moderate-stable | 2.36 (1.57, 3.56) |
| High-stable | 2.84 (1.64, 4.91) |
| **Myocardial infarction** |  |
| PAC trajectories |  |
| Low-stable | 1.00 (ref) |
| Moderate-stable | 1.96 (1.18, 3.27) |
| High-stable | 2.11 (1.07, 4.16) |

Model 5: model 4 further adjusted for the baseline PAC.

**Table S9**. E-values for the observed associations between PAC trajectories and clinical outcomes.

| Exposure | PAC trajectories | | |
| --- | --- | --- | --- |
|  | Low-stable | Moderate-stable | High-stable |
| **Cardiovascular disease** |  |  |  |
| Model 4 | 1.00 (ref) | 2.19 (1.59, 3.01) | 2.56 (1.68, 3.91) |
| E-value for point estimate | - | 3.80 | 4.56 |
| **Total stroke** |  |  |  |
| Model 4 | 1.00 (ref) | 2.36 (1.57, 3.56) | 2.84 (1.64, 4.91) |
| E-value for point estimate | - | 4.15 | 5.13 |
| **Myocardial infarction** |  |  |  |
| Model 4 | 1.00 (ref) | 1.97 (1.18, 3.28) | 2.29 (1.17, 4.46) |
| E-value for point estimate | - | 3.35 | 4.01 |

## 2.2 Supplementary Figures


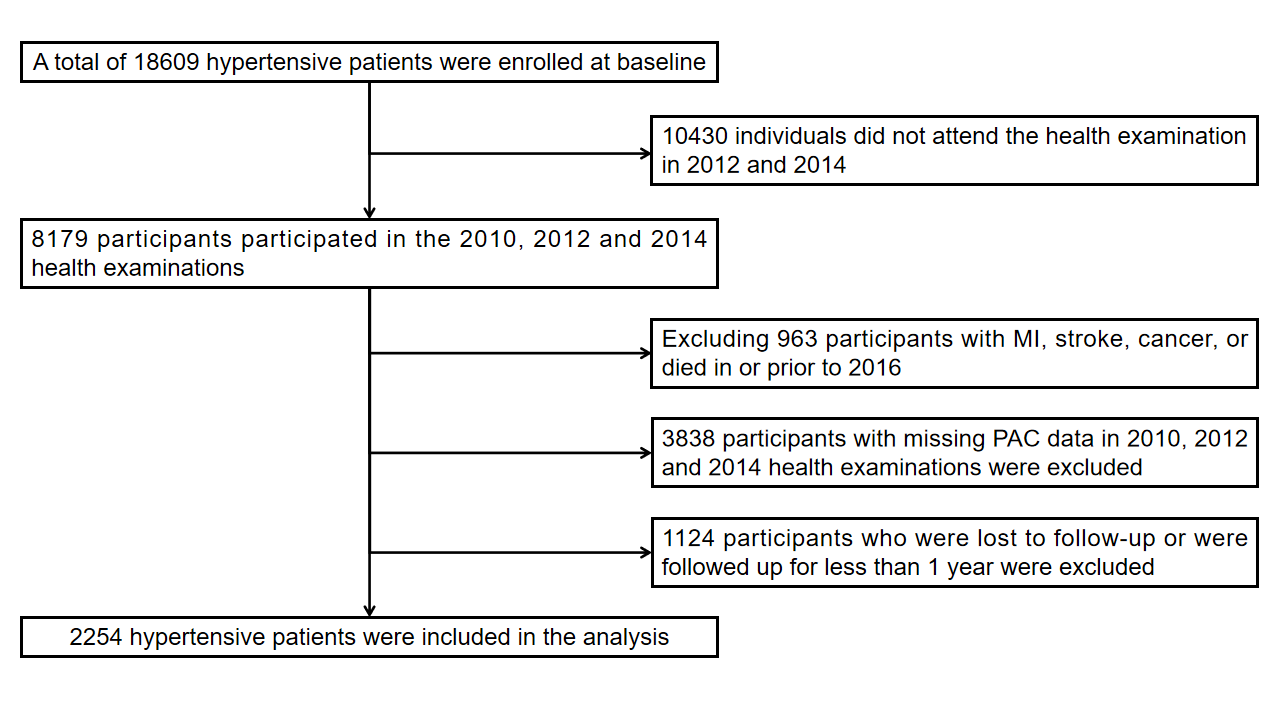


**Figure S1** Flowchart of the study.

**References**

[1] 2016 Chinese guidelines for the management of dyslipidemia in adults. J Geriatr Cardiol. 2018. 15(1): 1-29.

[2] Funder JW, Carey RM, Mantero F, et al. The Management of Primary Aldosteronism: Case Detection, Diagnosis, and Treatment: An Endocrine Society Clinical Practice Guideline. J Clin Endocrinol Metab. 2016. 101(5): 1889-916.

[3] Zhu Q, Heizhati M, Lin M, et al. Higher Plasma Aldosterone Concentrations Are Associated With Elevated Risk of Aortic Dissection and Aneurysm: a Case-Control Study. Hypertension. 2022. 79(4): 736-746.

[4] Tunstall-Pedoe H, Kuulasmaa K, Amouyel P, Arveiler D, Rajakangas AM, Pajak A. Myocardial infarction and coronary deaths in the World Health Organization MONICA Project. Registration procedures, event rates, and case-fatality rates in 38 populations from 21 countries in four continents. Circulation. 1994. 90(1): 583-612.

[5] Stroke--1989. Recommendations on stroke prevention, diagnosis, and therapy. Report of the WHO Task Force on Stroke and other Cerebrovascular Disorders. Stroke. 1989. 20(10): 1407-31.

[6] Wu S, An S, Li W, Lichtenstein AH, Gao J, Kris-Etherton PM, et al. Association of Trajectory of Cardiovascular Health Score and Incident Cardiovascular Disease. *JAMA Netw Open*. (2019) 2:e194758. doi: 10.1001/jamanetworkopen.2019.4758
